# Erratum

**DOI:** 10.1590/S1415-475738320140231e

**Published:** 2015

 Erratum to: Prevalence of b^S^-globin gene haplotypes, a-thalassemia (3.7 kb deletion) and redox status in patients with sickle cell anemia in the state of Paraná, Brazil. Eliana LitsukoTomimatsu Shimauti, Danilo Grunig Humberto Silva, Eniuce Menezes de Souza, Eduardo Alves de Almeida, Francismar Prestes Leal and Claudia Regina Bonini-Domingos. Genetics and Molecular Biology 38(3), 316-323.

The authors noted errors in certain values presented in Tables 3 and 4 of this paper due to misplacement of decimal points.

In Table 3 the values reading:

**Table:** 

Leukocytes (x10^9^/L)	12,600^a^(8,600-18800)	7,650^b^(4,300-10,900)	6,550^b^(3,800-10,500)
Neutrophils (x10^9^/L)	6,090^a^(4,472-1,1280)	4,002^b^(2,250-6,534)	3,454^b^(1,504-7,144)
Monocytes (x10^9^/L)	0.725^a^(0.172-2256)		

Should read:

**Table:** 

Leukocytes (x10^9^/L)	12.6^a^(8.6-18.8)	7.65^b^(4.3-10.9)	6.55^b^(3.8-10.5)
Neutrophils (x10^9^/L)	6.09^a^(4.472-1.128)	4.002^b^(2.25-6.534)	3.454^b^(1.504-7.144)
Monocytes (x10^9^/L)	0.725^a^(0.172-2.256)		

In Table 4 the values reading:

**Table:** 

Leukocytes (x10^9^/L)	12,150(8,800-18,800)	13,100(8,600-15,500)	NS
Neutrophils (x10^9^/L)	6,894(4,536-11,092)	5,633(4,386-8,370)	NS
Monocytes (x10^9^/L)	1,202(0.54-2,2256)	0.655(0.172-1,085)	0.03

Should read:

**Table:** 

Leukocytes (x10^9^/L)	12.15(8.8-18.8)	13.1(8.6-15.5)	NS
Neutrophils (x10^9^/L)	6.894(4.536-11.092)	5.633(4.386-8.37)	NS
Monocytes (x10^9^/L)	1.202(0.54-2.2256)	0.655(0.172-1.085)	0.03

